# A Case of Hydrophobia

**Published:** 1877-04

**Authors:** C. L. Edwards

**Affiliations:** Hyde Park


### A Case of Hydrophobia.

By C. L. Edwards, M. D., Hyde Park.

Miss A. B. was bitten last August, between the fore and middle fingers of her right hand, by a small black-and-tan terrier dog. The injury was so slight that she did not send for medical aid, but sucked the wound, after which she applied Friar's balsam and pork rind. It healed rapidly and she thought nothing more about it; the dog was killed to satisfy popular superstition, but was not supposed to be rabid. On February 14, 1877, while washing the tea things in warm water she suddenly felt a "sharp, stinging pain" at the seat of injury, which during the evening and night extended up the arm to the shoulder. The next morning there was great difficulty in swallowing and a feeling of constriction in the neck and upper part of the chest. She being then at Norwood sent for Dr. Fogg, who immediately recognized the disease and advised removal to her home in Hyde Park, where she arrived at four P. M. the same day. I saw her at five P. M. She was then on a lounge, her tongue clean, skin cool, pulse 95 and hard. There was nothing unusual about her appearance. I noticed that when answering questions she spoke during the act of inspiration. Otherwise she was calm and tranquil, but on offering her some water from a tea spoon the true symptoms presented themselves; at sight of it severe contraction of the muscles of the throat occurred, accompanied by a sort of spasmodic sobbing, which as the spoon approached her lips was fearfully increased. She tried bravely to take the water, but, with the exception of a few drops after great exertion, it was impossible. At my suggestion she went to bed; I gave her one sixth of a grain of sulphate of morphine subcutaneously, ordered hot bricks to her feet, and injections of beef tea and brandy into the rectum *pro re nata;* perfect quiet was enjoined.

At three o'clock the next morning I was called, the messenger telling me that the poor girl was suffering terribly. I found her on her back, the hands clutching at the throat and chest, with severe spasms of muscular contraction; the pulse was 120; the skin very hot and moist; she had passed urine and retained two injections of beef tea and brandy, which had been given as ordered. A quarter of a grain of sulphate of morphine injected into the arm gave her comparative ease, but no sleep. I went home at six A. M. Accompanied by Dr. W. S. Everett, of Hyde Park, I saw her at ten A. M.; she was tolerably quiet when all the surroundings were still, but the opening of a door or the rustling of a dress would immediately bring on the spasms, and the attempt at swallowing was so painful that we thought it best to abandon it altogether. Her only complaint was that her head was dizzy and she felt "so, so tired;" pulse 120.

I saw her at one P. M., in consultation with Dr. C. C. Holmes, of Milton. The symptoms were rather more aggravated. She was ordered tincture of aconite, chloroform and alcohol to spine, and morphine sulphate one sixth grain, with chloral hydrate grs. v. subcutaneously as occasion required. During the afternoon she gradually grew worse, her urine passed involuntarily, aud the throat got very dry and parched; she would make violent unsuccessful efforts to vomit, coughing frequently spitting out a thick bloody mucus, which she would take in her fingers and pull from her mouth, not being able to permit even the approach of a handkerchief to her face. "Water," was now her cry, "give me water," and so eager was she to relieve the dryness of her throat, that some water would actually be swallowed before she seemed to be aware of what she had done, and then the spasmodic choking would come on more severely.

During the night following, she had short periods of comparative ease, but the end seemed surely approaching. When the spasms now occurred they became more general, the body and lower extremities being terribly convulsed, so much so that it required half a grain of morphine once an hour for three successive hours to afford her any relief, and that was but little The pulse became intermittent, ranging from 150 to 160. For two hours previous to death, which took place at 9.30 A. M., she was almost free from spasms, and she talked glibly of things which happened a year or two before, going through minutely the history of her dog bite. A little after nine A. M. she was seized with a severe spasm, and died asphyxiated in less than half an hour.

There were no attempts made at any time during her sickness to bite or to bark like a dog, but there was a very harsh dry cough which I can easily imagine that the ignorant might have conjured into a bark. It was just sixty-two hours from the time she first felt the pain in her hand till death ensued.—*Boston Med. Jour.*

